# Normal sonographic liver and spleen dimensions in a central European pediatric population

**DOI:** 10.1186/s12887-021-02756-3

**Published:** 2021-06-11

**Authors:** Stephan Waelti, Tim Fischer, Simon Wildermuth, Sebastian Leschka, Tobias Dietrich, Sabine Guesewell, Pascal Mueller, Michael Ditchfield, Stefan Markart

**Affiliations:** 1 Department of Radiology and Nuclear Medicine, Children’s Hospital of Eastern Switzerland, Claudiusstrasse 6, 9006 St. Gallen, Switzerland; 2 Department of Radiology and Nuclear Medicine, Cantonal Hospital St. Gallen, Rorschacher Strasse 95, 9007 St. Gallen, Switzerland; 3 University of Zurich, Faculty of Medicine, Pestalozzistrasse 3, 8091 Zurich, Switzerland; 4 Cantonal Hospital St. Gallen, Clinical Trials Unit, Biostatistics, Bedastrasse 1, 9000 St. Gallen, Switzerland; 5 Division of Pediatric Gastroenterology and Hepatology, Children’s Hospital of Eastern Switzerland, Claudiusstrasse 6, 9006 St. Gallen, Switzerland; 6 Department of Diagnostic Imaging, Monash Health, Monash Children’s Hospital, 246 Clayton Road, Clayton, 3168 Australia

**Keywords:** Liver size, Spleen size, Ultrasound, Children, Caucasian

## Abstract

**Background:**

Organ size is influenced by a number of factors. Age, height, weight, and ethnicity are known influencing factors. Pediatric populations have changed over time, puberty beginning earlier resulting in a changing growth pattern of their organs. Hence, contemporary charts using local data are considered the most appropriate for a given population. Sonographic charts for liver size for a predominantly Caucasian population are limited, which has implications for clinical practice. The aim of this study was to define a contemporary normative range of liver and spleen sizes for a healthy, predominantly Caucasian population and for all pediatric age groups (0–18 years) and to investigate whether there is a size difference between genders and ethnicities.

**Methods:**

Retrospective study including children with normal sonographic findings and no evidence of liver or splenic disease clinically. Craniocaudal and anteroposterior dimensions are measured for the right and left lobe of the liver, and craniocaudal dimension for the spleen. Relationship of the liver and spleen dimensions with age, body length, body surface area, weight, and gender were investigated. Charts of normal values were established. Values were compared to studies involving other ethnicities and to one study carried out in 1983 involving the same ethnicity.

**Results:**

Seven hundred thirty-six children (371 boys, 365 girls) aged 1 day - 18.4 years were included. From the second year of life, the craniocaudal dimension of the right lobe of the liver is 1–2 cm larger in the Central European population compared with non-Caucasian populations at a given age. Liver size of Central European children in 2020 is greater compared to a similar population almost 40 years ago. The craniocaudal dimension of the spleen of Central European, US-American and Turkish children is similar. The difference between genders is statistically significant for both the liver and the spleen, being larger in boys.

**Conclusion:**

Contemporary and ethnically appropriate reference charts for liver and spleen measurements should be used, especially for liver size. The effect of ethnicity is reduced if patient height rather than age is referenced.

## Background

Abdominal ultrasound is routinely used in pediatric patients in the investigation of many diseases and to measure and monitor normal organ growth [1, 2]. The size of the liver and spleen can provide important clues about the presence of certain pathologies. Accordingly, referring clinicians frequently ask for liver and spleen size, even though the sonographic measurement of liver size in particular has certain limitations.

Organ size is influenced by a number of factors. Age, height, weight, body surface area and ethnicity are known influencing factors and has been well documented for the kidneys [3–7]. Hence, charts using local data are considered the most appropriate for a given population [3, 8].

With changes in nutrition and disease profiles, pediatric populations have also changed over time, puberty beginning earlier resulting in a changing growth pattern of their organs. The average age of menarche in girls declined over the past century from an average age of 16–17 years to 12–13 years [9, 10]. American boys reach puberty 6 months to 2 years earlier than just a few decades ago [11]. Therefore, the use of contemporary charts is important. The timing of puberty also differs between ethnicities. African American boys commence puberty the earliest, at around 9 years of age, while Caucasians and Hispanic boys commencing on average at age 10 years [11]. This further emphasizes the importance of using charts based on data from the same ethnicity.

Only a limited number of contemporary studies of normative sonographic liver size in children have been published and include a Turkish population aged 0–16 years in 1998 [2], a Brazilian population aged 0–7 years in 2009 [12], an Indian population aged 0–12 years in 2010 [13], a Nepalese population aged 0–15 years in 2014 [5] and a second Nepalese population aged 0–15 years in 2015 [6]. Sonographic charts for liver size for a predominantly Caucasian population are limited. To our knowledge, only one such study exists, published in 1983 with 194 German children aged 0–18 years [14].

The lack of contemporary normative charts has implications for clinical practice: At our pediatric hospital in Central Europe with predominantly Caucasian patients, using the available reference charts, we have found a high frequency of hepatomegaly in the absence of other findings of liver disease. This may lead to unnecessary hepatological and/or hematological consultations and laboratory tests, with associated unnecessary cost and uncertainty for the affected families.

The aim of this study was to define a contemporary normative range of liver and spleen sizes for a predominantly Caucasian population and for all pediatric age groups (0–18 years) in correlation with age, height, weight and body surface. We also investigated the influence of gender.

## Methods

Organ sizes are routinely measured at our hospital during each abdominal sonography. The measurements are made during breath-holding in older children and during quiet breathing in younger children.

**Fig. 1:**
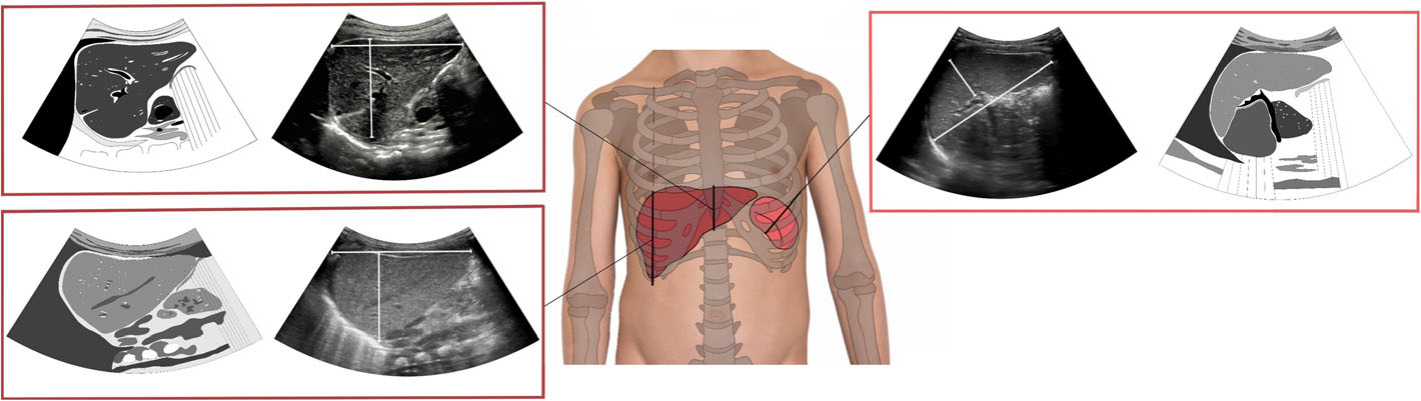
Schematic representation showing the planes and reference points for liver and spleen measurements. Left liver lobe plane is established by the midsternal line and right liver lobe plane by the midclavicular line. Spleen measurements are performed in the craniocaudal course of the intercostal spaces at the level of the splenic hilum. Measurements are performed between the most superomedial and the most inferolateral points of the spleen. The transverse diameters are measured at right angles to the craniocaudal measurements

For liver and spleen size, we have based our measurement technique on the publication by Konus et al. [2]. We strictly adhere to this standardized technique in everyday clinical practice enabling comparability in follow-up examinations. Liver measurements are performed in the supine position, based on external orientation lines. Craniocaudal and anteroposterior dimensions are obtained in the midclavicular plane for the right liver lobe and midsternal plane for the left liver lobe. Midsternal plane means that the plane passes through the xiphoid process. Craniocaudal diameters are measured in a horizontal line parallel to the abdominal wall (Fig. 1). In both planes, the upper margin of the liver is defined as the uppermost edge under the dome of the diaphragm, whereas the lower margin is defined as the lowermost edge of the lobe. A short segment of the most cranial part of the liver is sometimes not visible due to the acoustic shadowing of the lung, especially in the right liver lobe. If despite the use of convex probes this problem persists, we extrapolate the assumed curvature of the upper edge of the liver and perform the measurement. The anteroposterior diameter is measured at a perpendicular angle to the craniocaudal measurement. The midclavicular plane is always used, even if the right lobe of the liver reaches far inferiorly (ie. so-called Riedel lobe).

Spleen measurements are performed in the supine position. The ultrasound probe is positioned in the craniocaudal course of the intercostal spaces at the level of the splenic hilum. Measurements are performed at the visually maximal distance between the most superomedial and inferolateral points of the spleen. The splenic transverse diameter is measured at right angles to this at the level of the hilum from the medial to the lateral edge (Fig. 1). Accessory spleens were not included in the measurements.

The organs were measured on a Philips IU22 (Philips Healthcare, Best, the Netherlands) or GE Logic E9 (GE Healthcare, Waukesha, WI) ultrasound systems. Measurements were performed by one of two pediatric radiologists with 10 and 19 years of experience, personally or by one of five registrars with 1–5 years of experience. The registrars’ measurements were each reviewed during the examination by one of the two pediatric radiologists and corrected if necessary.

We retrospectively searched our RIS/PACS system for pediatric patients aged 0–18 years who had undergone abdominal sonography between May and September 2020 and had no pathologic findings related to the liver or spleen, neither clinically nor sonographically. The radiological reports were screened for the presence of normal findings for the liver and spleen. The clinical data was screened for the presence of diseases that may affect the liver and spleen size which included cystic fibrosis, hepatitis, liver steatosis, hemoglobinopathies, oncologic diseases (e. g. leukemia), cardiopathies, previous liver surgery, portal vein pathology, scoliosis, growth disorders, chromosomal anomalies, syndromic diseases and drugs. The clinical data was also screened for pathological liver enzymes and cholestasis parameters. If any of these abnormalities were present, the child was excluded from the study. Premature births and twins/multiple pregnancies were excluded until the age of 2 years. Older, former premature infants and twins were included in the study using the chronological age. Children with urinary tract infection were included. If several abdominal ultrasounds were performed on a child during the study period, only the first examination was included. All ethnic groups were included in the study and non-Caucasians were not excluded.

We then reviewed the image quality and excluded patients if all hepatic and splenic measurements were not performed or it was considered of poor quality (e.g., due to movement artifact or abdominal gas distention or acoustic shadowing of the lung prevented reliable measurements). For the normal echogenicity of the liver, we used the echogenicity of the right renal cortex as a reference. Patients with pathological echogenicity of the liver parenchyma or an abnormal position of the organs were excluded. The patients age at examination, gender, height, and weight was recorded within the RIS/PACS system.

Ethics approval was obtained from the local Ethics committee.

### Statistical analysis

All processing and analysis were carried out with the software R, Version 4.0.2. Children were subdivided into age groups based on their age in days. The main measures of interest, i.e., right liver lobe craniocaudal diameter and spleen length, were summarized in tables of descriptive statistics by age group after checking that they were normally distributed within age groups. The dependence of these organ size measures on age and on body size was further described with Spearman rank correlations and represented graphically using scatter plots with smoothing lines for the 5th, 50th, and 95th percentiles obtained through additive quantile regression.

Because earlier studies suggested a decreasing rate of organ growth with age (faster growth in very young children), the presence of a non-linearity in the relationship between organ size and age was tested with linear modes that included a quadratic term.

To compare organ dimensions between genders, generalized additive models including gender and a smoothing term for age were fitted to obtain an estimate of the mean difference between boys and girls at the same age. We also fitted models including a second smoothing term for height to see whether differences in organ size persisted after adjusting for differences in body size.

To compare age-dependent organ sizes in our study population with those of children in other world region, tables of summary statistics for right liver lobe craniocaudal diameter and spleen length by age group, including the minimal and maximal age of each age group, mean organ size, and the standard deviation, were extracted from publications. These summary measures were chosen because they were given in all publications. The US study [15] provided data separately for boys and girls. These were pooled using weighted means of each gender’s mean and variance (squared SD). Mean age per group was calculated as the mean of the group’s age range. Mean organ size was plotted against mean age for each study population. Mean liver size was also plotted against mean height (or mean of the age group’s height range) to determine whether differences in liver size were associated with differences in body size.

## Results

2133 abdominal sonographies were performed during the study period. Of these, 1397 (65.5%) sonographies were excluded for clinical reasons, pathological hepatic or splenic findings, inadequate measurement, incomplete data (missing patient height or weight) or for multiple abdominal sonographies performed on the same child. Ultimately, the study population consisted of 736 abdominal sonographies from 736 children (371 (50.4%) boys and 365 (49.6%) girls). The age ranged from full-term newborn (1 day) to 18.4 years (median 7.3 years). The median height was 123 cm (range 43–196 cm), weight 25 kg (2–96 kg), body surface area [16] 0.92 m^2^ (0.15–2.28 m^2^) and BMI: 16.6 kg/m^2^ (11.2–34.8 kg/m^2^). The patient population of our hospital consists of approximately 95% Caucasians, 2% Asians, 1% Africans and 2% others.

### Liver

Descriptive statistics for the right lobe craniocaudal diameter within each age group are given in Table 1.

**Table 1:** Right liver lobe craniocaudal diameter

Age group	Height	n	Mean	SD	Minimum	Maximum	Percentiles (cm)	
	range (cm)		(cm)	(cm)	(cm)	(cm)	5th	95th
0–1 mo*	47–55	22	6.2	0.95	4.7	8	5.2	8
> 1–6 mo	43–72	49	6.8	0.92	4.6	8.4	5.2	8.3
> 6–12 mo	53–86	45	7.9	1.34	4.1	10.5	5.4	10.0
> 1–2 yr	71–97	54	8.4	1.27	5.4	11.4	6.2	10.3
> 2–3 yr	70–110	33	8.8	1.16	6.4	11.1	7.1	10.8
> 3–4 yr	91–119	30	9.6	0.90	7.1	11.2	8.3	11.0
> 4–5 yr	90–112	34	9.9	0.85	7.7	11.8	8.8	11.2
> 5–6 yr	103–128	53	10.5	1.13	7.3	13.0	8.9	12.1
> 6–7 yr	109.5–142	39	10.9	1.17	8.8	13.4	9.2	13.0
> 7–8 yr	110–144	45	11.0	1.24	8.5	13.6	9.2	12.7
> 8–9 yr	121–148	46	12.0	1.40	9.0	15.2	9.9	14.8
> 9–10 yr	121–164	34	12.0	1.34	9.6	15.8	10.2	13.6
> 10–11 yr	123–162	42	12.2	1.10	10.1	16.0	10.5	13.7
> 11–12 yr	82–160	35	12.2	1.47	8.2	15.6	10.2	14.3
> 12–13 yr	94–181	41	12.5	1.59	7.2	16.5	10.5	14.8
> 13–14 yr	142–171	32	13.5	1.21	11.6	15.8	11.9	15.6
> 14–15 yr	149–178.5	40	13.7	1.32	11.2	18.0	11.6	15.8
> 15–16 yr	153–196	28	13.4	1.95	8.7	16.2	9.4	15.9
> 16–18 yr	149–183.2	34	14.3	1.36	11.7	17.0	12.2	16.4

Descriptive statistics for right liver lobe craniocaudal diameter within each age group

**Fig. 2:**
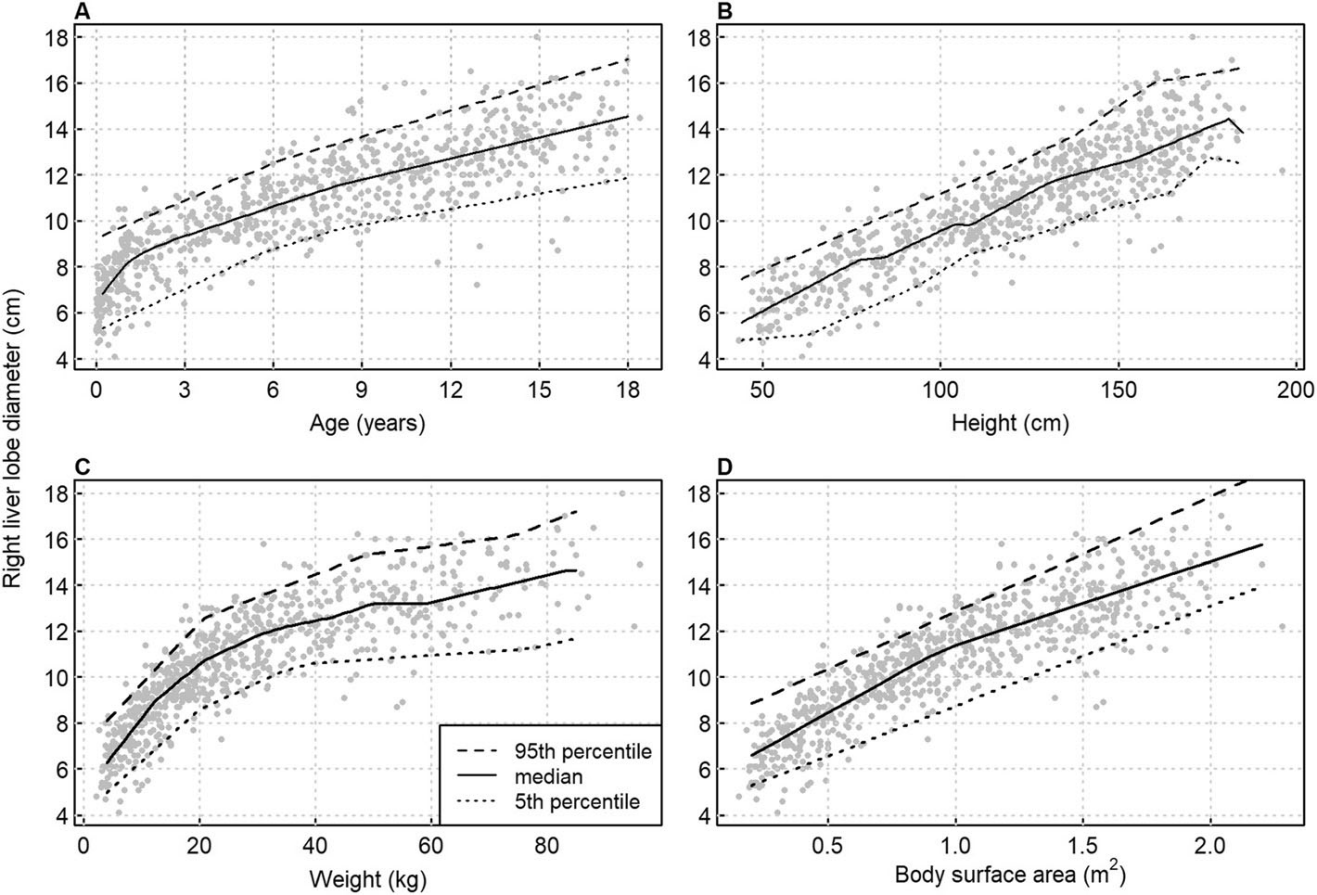
Scatter plots of right liver lobe craniocaudal diameter against (**A**) age, (**B**) body height, (**C**) weight and (**D**) body surface area, with smoothing lines for the 5th, 50th, and 95th percentiles, obtained through quantile regression

There was a non-linear relationship between the craniocaudal diameter of the right lobe and age or weight (Fig. 2A, C). The relationship of liver size with body height and BSA was more linear (Fig. 2B, D). The non-linearity of the increase in liver size with age was statistically significant (*p* < 0.001) for both dimensions of the right lobe and for the craniocaudal dimension of the left lobe. For the anteroposterior dimension of the left lobe, the non-linearity was not statistically significant (*p* = 0.10). For the right lobe’s craniocaudal dimension, the non-linearity implies a mean growth rate of 1 cm/year until 3 years but only 0.33 cm/year from 3 to 18 years.

At any given age or body height, the range of normal values for right-lobe craniocaudal dimension was approximately 4 cm, or mean ± 2 cm (Fig. 2A, B, C, D). The association with weight became weak beyond 30 kg (Fig. 2C), reflecting both the decreasing growth rate with age and the weak relationship between organ size and BMI.

Correlations between liver dimensions and age or body size were generally stronger for the right lobe than for the left lobe, and stronger for the craniocaudal than for the anteroposterior dimension (Table 2).

**Table 2:** Correlations between liver diameters and age or body size

Body	Right liver lobe diameter		Left liver lobe diameter	
measure	craniocaudal	anteroposterior	craniocaudal	anteroposterior
Age	0.87 (0.85 to 0.89)	0.81 (0.78 to 0.83)	0.75 (0.71 to 0.78)	0.64 (0.59 to 0.68)
Height	0.89 (0.87 to 0.90)	0.83 (0.80 to 0.85)	0.75 (0.71 to 0.78)	0.65 (0.60 to 0.69)
Weight	0.88 (0.86 to 0.90)	0.85 (0.82 to 0.87)	0.73 (0.69 to 0.77)	0.68 (0.63 to 0.72)
BSA^a^	0.89 (0.87 to 0.90)	0.85 (0.82 to 0.87)	0.74 (0.70 to 0.77)	0.67 (0.63 to 0.71)
BMI^b^	0.44 (0.38 to 0.50)	0.51 (0.45 to 0.57)	0.29 (0.23 to 0.36)	0.46 (0.40 to 0.52)

Spearman rank correlations between liver size and age or body size with 95% confidence intervals obtained through Fisher’s transformation.

^a^BSA (Body Surface Area), ^b^BMI (Body Mass Index)

The difference between genders was statistically significant (*p* < 0.05) for both dimensions of the right liver lobe and for the anteroposterior dimension of the left liver lobe. For these dimensions, the liver diameter of girls was on average between 0.17 and 0.24 cm smaller than that of boys. The difference in liver size between genders could be partly, but not fully, explained by the fact that girls were generally smaller than boys (mean height difference at the same age: − 1.3 cm, mean weight difference: - 0.61 kg). If body height was included in the generalized additive models, differences between genders were reduced by 25–30% and no longer statistically significant (*p* > 0.05, Table 3).

**Table 3:** Difference of liver diameters between genders

Model covariates	Age		Age and height	
	diff (cm)	*p*-value	diff (cm)	*p*-value
Right lobe craniocaudal	−0.24	0.012	−0.16	0.070
Right lobe anteroposterior	−0.21	0.018	−0.16	0.067
Left lobe craniocaudal	−0.09	0.288	−0.07	0.403
Left lobe anteroposterior	−0.17	0.012	−0.13	0.055

Difference between genders as determined from models including smoothing terms for the covariates (1) age, (2) age and height. The mean difference between genders (females compared with males) as well as the significance of this difference are given for each liver dimension

### Spleen

Descriptive statistics for spleen length within each age group are given in Table 4.

**Table 4:** Spleen length

Age group	Height	n	Mean	SD	Minimum	Maximum	Percentiles (cm)	
	range (cm)		(cm)	(cm)	(cm)	(cm)	5th	95th
0–1 mo	47–55	22	4.8	0.6	3.5	6.3	4.1	6
> 1–6 mo	43–72	49	5.7	0.9	3.2	7.4	4.4	6.8
> 6–12 mo	53–86	45	6.2	0.8	4.8	7.8	5.2	7.7
> 1–2 yr	71–97	54	6.6	0.8	4.8	8.8	5.5	8.2
> 2–3 yr	70–110	33	7.4	1.0	4.5	9.0	6.0	8.9
> 3–4 yr	91–119	30	7.5	0.8	6.2	9.7	6.4	8.7
> 4–5 yr	90–112	34	8.1	0.9	6.2	10.6	6.9	9.3
> 5–6 yr	103–128	53	8.2	0.9	5.9	10.4	6.7	9.6
> 6–7 yr	109.5–142	39	8.4	0.8	6.3	10.0	7.4	9.6
> 7–8 yr	110–144	45	8.7	1.2	5.4	11.4	6.8	10.3
> 8–9 yr	121–148	46	9.2	0.9	7.5	11.7	7.9	10.5
> 9–10 yr	121–164	34	9.4	1.1	7.8	12.2	8.0	11.6
> 10–11 yr	123–162	42	9.8	1.2	7.4	13.0	8.3	12.0
> 11–12 yr	82–160	35	9.9	1.4	6.4	13.3	7.8	11.7
> 12–13 yr	94–181	41	10.2	1.1	7.2	12.5	8.8	12.0
> 13–14 yr	142–171	32	10.2	1.3	8.1	12.7	8.2	12.3
> 14–15 yr	149–178.5	40	10.5	1.1	8.6	13.0	8.8	12.2
> 15–16 yr	153–196	28	10.5	1.2	6.9	12.6	8.7	12.2
> 16–18 yr	149–183.2	34	10.9	1.4	8.1	14.3	8.8	13.4

Descriptive statistics for spleen length within each age class

**Fig. 3:**
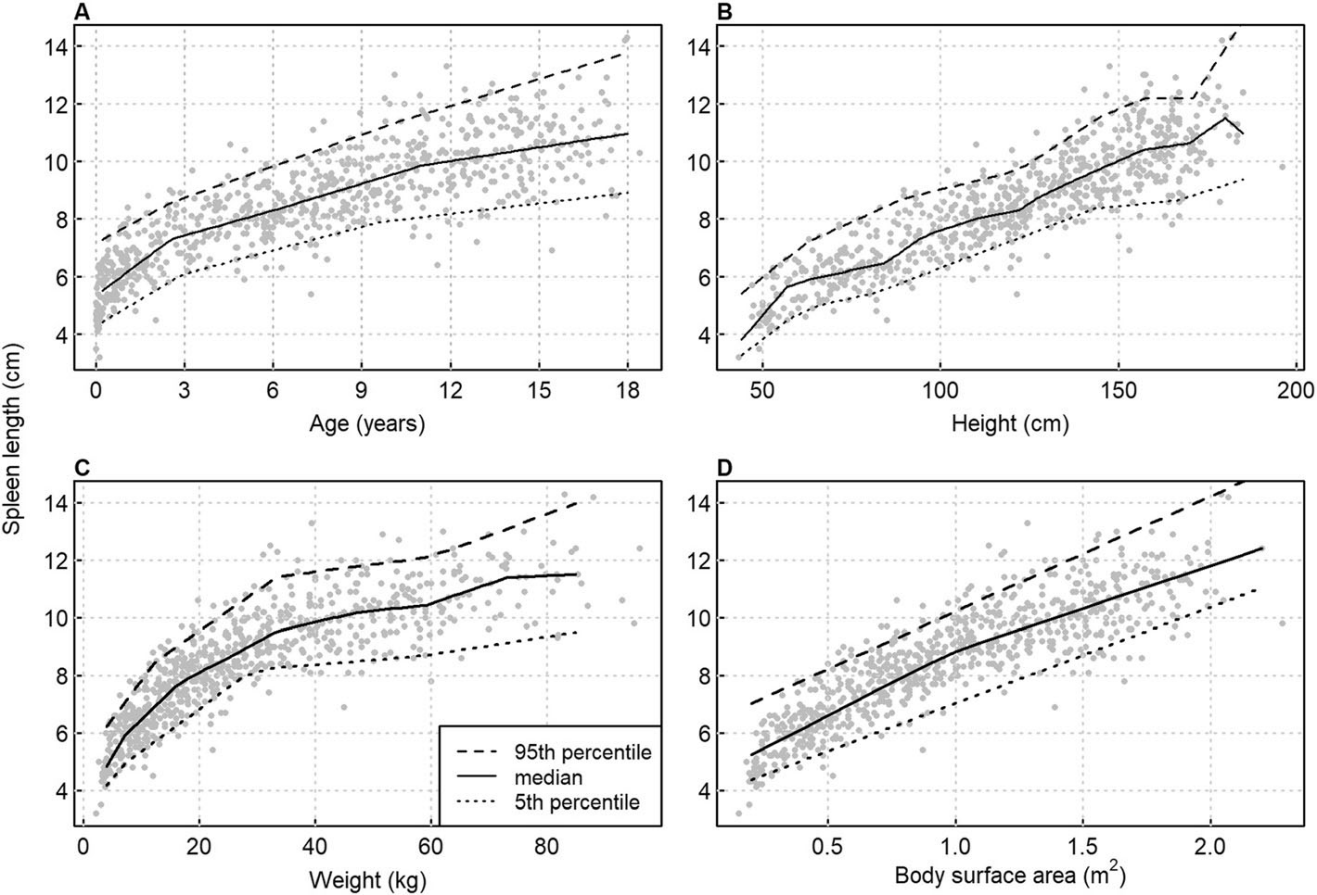
Scatter plots of spleen length against (**A**) age, (**B**) body height, (**C**) weight and (**D**) body surface area, with smoothing lines for the 5th, 50th and 95th percentiles, obtained through quantile regression

There was a non-linear relationship between both spleen dimensions and age (Fig. 3A) or weight (Fig. 3C). The mean increase in spleen length with age was 0.79 cm/year until 3 years and 0.24 cm/year above 3 years. The mean growth in transverse dimension with age was 0.25 cm/year until 3 years and 0.09 cm/year above 3 years. The relationship of spleen size with body height was essentially linear (Fig. 3B). Quantile regression indicates a normal range of approximately 4 cm in young children, which widened up to 6 cm in the oldest children (Fig. 3A).

There was a significant difference between genders for both dimensions of spleen size. At the same age, girls had spleens on average 0.24 cm smaller in longitudinal dimension and 0.17 cm smaller in transversal dimension than boys. The gender difference in transversal dimension increased significantly with age (*p* = 0.013 for the age*gender interaction). The gender difference was slightly reduced if body height was taken into account (females were then 0.19 and 0.14 cm smaller in the two dimensions), but still highly significant (*p* < 0.001). This was also true if body weight was additionally taken into account.

## Discussion

Patient demographic (age) and morphometric (height, weight) factors have been shown to influence liver and spleen size [2, 5, 6, 12–15, 17–19]. As these factors obviously change during childhood, normative data on organ size in children is of great significance for differentiating between a normal and pathological condition. Only a few charts are available for sonographic liver size in children. These charts were not established with a mainly Caucasian population. The available, more recent main studies include a Turkish population aged 0–16 years in 1998 (307 patients) [2], a Brazilian population aged 0–7 years in 2009 (584 patients) [12], an Indian population aged 0–12 years in 2010 (597 patients) [13], a Nepalese population aged 0–15 years in 2014 (225 patients) [5] and a second Nepalese population aged 0–15 years in 2015 (272 patients) [6]. As far as we know, the only Caucasian study was published in 1983 with 194 German children aged 0–18 years [14]. The need for a chart with mainly Caucasian patients is based on the premise that organ size may be ethnicity-dependent. Ethnical differences in renal size have been shown between African-American and Caucasian children [20], between Hong Kong children and Western children [3], and between Australian aboriginal and non-Aboriginal children [8].

**Fig. 4:**
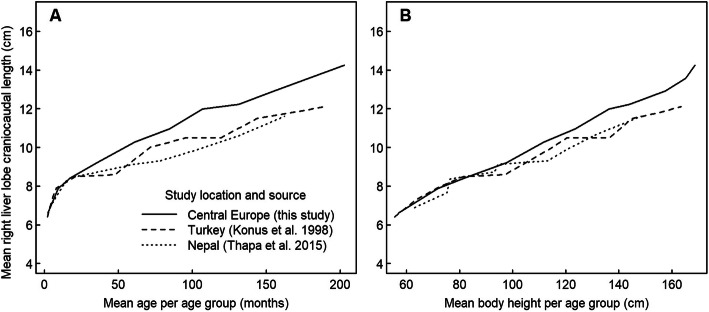
Mean right liver lobe craniocaudal diameter by age group (**A**) and by body height (**B**) in the Central European population studied here and in Turkish and Nepalese populations based on published data. For each age group, liver dimensions are plotted against the mean of the age range (**A**) and the mean of the height range (**B**) included in the group

The mean right liver lobe craniocaudal dimension was initially similar in the Central European, Turkish and Nepalese populations but diverged from the second year of life of the children, being 1–2 cm larger in the Central European population at a given age (Fig. 4A). Standard deviations within age groups were similar in the Central European and Turkish populations (on average 1.28 and 1.16 cm, respectively) and smaller in the Nepalese population (0.86 cm). The difference between world regions was slightly reduced, but not eliminated, if the right liver lobe craniocaudal dimension was related to body height (Fig. 4B). Thus, for a given body height, children in Central Europe had slightly larger livers than children in Turkey and Nepal.

**Fig. 5:**
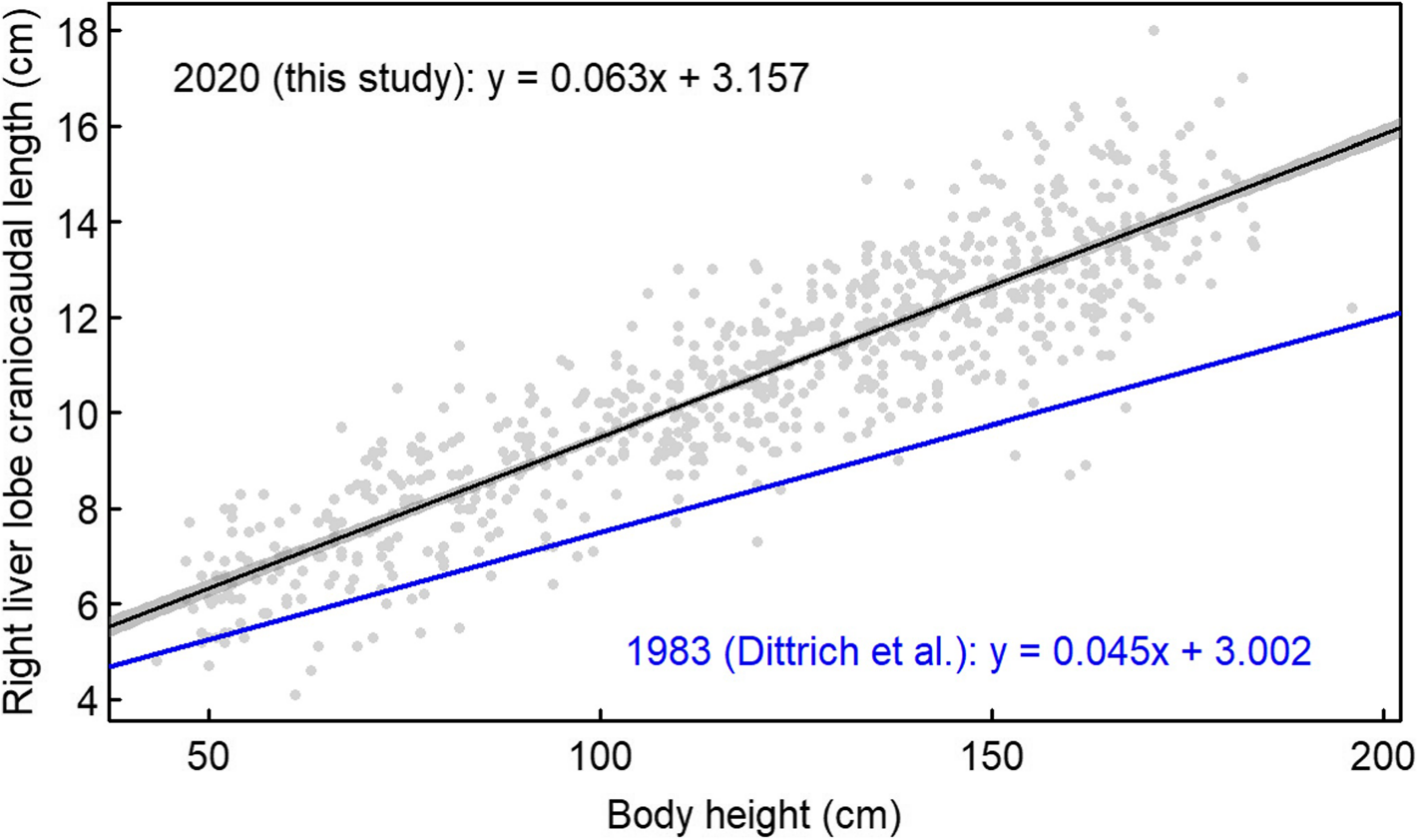
Linear relationship between body height and the right liver lobe craniocaudal diameter in the present study (regression line with 95% confidence band) and the regression line reported for children in Germany by Dittrich et al. [14]

By comparing our European study population with another European (Germany) study population from 1983 [14], the slope and the intercept of the linear relationship between body height and the right liver lobe craniocaudal dimension differed clearly between the present study and the regression line reported for German children in 1983 (Fig. 5). Therefore, even for a given body height, Central European children had a larger liver in 2020 than they had almost 40 years before.

Hence, according to our data, liver size also differs between ethnic groups, not only compared to older studies such as the 1998 Turkish study [2], but also compared to a recent 2015 Nepalese study population [6]. Comparison with the 1983 German study [14] shows that liver size has also changed over time within an ethnic group, possibly due to increasing energy intake and body size. It is possible that the difference with a current Turkish population would not be as great as it was with the 1998 Turkish population, as it is likely that the liver size of Turkish children has also changed over time. It can be concluded that for liver size, local and current reference charts should be used as far as possible.

The liver shape varies to some extent from one patient to another. The wide range of normal dimensions of our study and of previous studies reflects that. Nevertheless, it is important to have reference charts for normal liver dimensions in the pediatric age group to be a guide as to whether or not the liver size is normal. Liver diameters can be measured in several ways. In four of the aforementioned studies [2, 5, 12, 13] the size of the right liver lobe is measured in the midclavicular line. However, two of these studies [12, 13] corrected the image plane so that the right kidney was still well visualized. Calle-Torro et al. [17] have, in a review paper, attempted to compare the various methods of liver measurement and collate the data in varied age groups. Ultimately, the measuring is performed slightly differently in every trial, and the patients collated in different age groups, limiting the data analysis.

As in previous studies, the craniocaudal dimension of the right lobe of the liver demonstrated the strongest correlation with age and body size and is therefore the most useful practical measurement.

Similar to Konus et al. [2] and Dittrich et al. [14], we found that height and body surface area (BSA) had the strongest correlation with liver size. In view of its simplicity, we recommend using the patient height parameter.

Some publications have concluded that gender impacts on renal size [4, 20, 21] and we therefore evaluated the impact of gender on liver and spleen size. Konus et al. [2] did not find a statistically significant difference between the two genders in liver and spleen dimensions in any age group. This is not consistent with our results. Both the liver and spleen are statistically significantly larger in boys. However, we do not consider the difference significant at a clinical level and a chart of composite data simplifies the clinical application of the chart.

There is a greater number of publications, many of which are recent, for splenic size. These include studies of an American population aged 0–17 years in 2004 (454 patients) [15], an Indian population aged 0–12 years in 2010 (597 patients) [13], a Nepalese population aged 0–15 years in 2015 (272 patients) [6], and a Turkish population aged 0–16 years in 2018 (310 patients) [18].

**Fig. 6:**
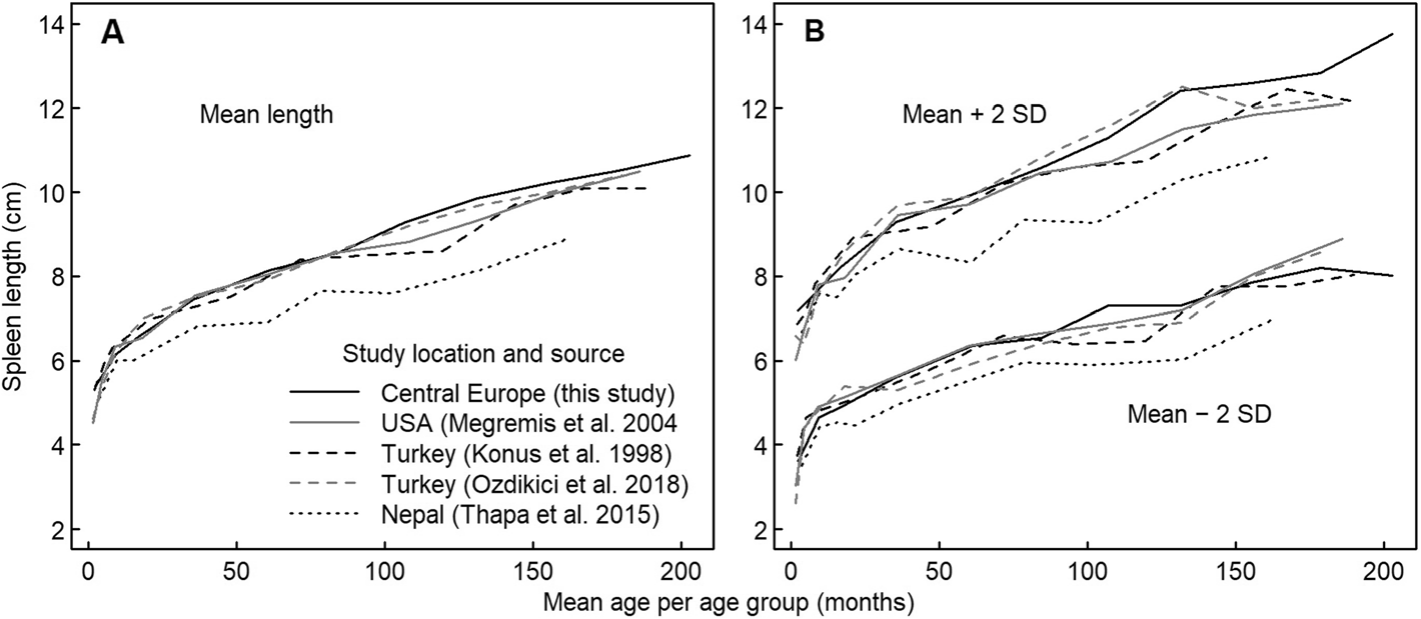
Mean spleen length by age group as well as approximate 5th and 95th percentiles (calculated as mean ± 2 SD) in the Central European population studied here and in three other populations based on published data. For each age group, spleen length is plotted against the mean of the age range included in the group

The spleen length of Central European, US-American and Turkish children was similar, whereas Nepalese children had smaller spleens (Fig. 6). The difference between world regions was reduced, but not eliminated, if spleen length was related to body height. Thus, for a given body height, children in Nepal had smaller spleens than children in Turkey and Central Europe. The difference between boys and girls at a certain age was slightly more pronounced and more consistent in the US-American study than in the present study, supporting the existence of gender differences.

When comparing ethnic groups, the difference for the spleen was less pronounced than for the liver; only Nepalese children had smaller spleens. We consider the use of a local reference chart to be appropriate, but not mandatory.

The best correlation with spleen size was shown with weight, BSA and height, with height and weight being the easiest to use in practice. There was also a significant difference in spleen size between the genders. As with the liver, we do not consider this difference relevant for everyday use.

In determining the presence of hepatic or splenic pathology, the dimensions of the organ are very relevant. Whether a routine measurement of the liver and spleen size should be carried out without evidence of a disease of these organs depends very much on the local practice and needs of the referring clinicians. Some clinicians will use organ size in a similar way to body length, head circumference or body weight, as a marker of normal development. However, routinely measuring organ size means that those with organs in the upper range of normal in size are at risk of being unnecessarily further investigated. We recommend measuring the size of the liver and spleen and comparing it with the reference charts if there are indications of a disease of these organs or if a disease should be excluded.

The retrospective design of this study is a limitation, since the measurements were not carried out by more than one radiologist and therefore no interobserver agreement could be determined. However, by having all measurements performed or checked by one of the two pediatric radiologists, we believe we have achieved a high level of standardization. A prospective setting in which all measurements for each patient are carried out by several radiologists and with the determination of the interobserver agreement should be the goal for a future study.

## Conclusion

We have established reference charts for pediatric liver and spleen dimensions correlating with age, weight and body length from a contemporary European, mainly Caucasian population and cover patients aged 0–18 years. Gender had a significant influence on organ size, but this difference was not felt to be relevant in daily clinical practice. Ethnicity has a greater influence on liver size compared to spleen size and therefore local reference charts should be used when available.

## Data Availability

The datasets used and/or analyzed during the current study are available from the corresponding author on reasonable request.

## Abbreviations

BMI: Body Mass Index
BSA: Body Surface Area
PACS: Picture archiving and communication system
RIS: Radiological Information System
SD: Standard Deviation
US: Ultrasound
